# The Good and the Bad: Monocytes’ and Macrophages’ Diverse Functions in Inflammation

**DOI:** 10.3390/cells11121979

**Published:** 2022-06-20

**Authors:** Judith Austermann, Johannes Roth, Katarzyna Barczyk-Kahlert

**Affiliations:** Institute of Immunology, University of Münster, 48149 Münster, Germany; judith.austermann@ukmuenster.de (J.A.); rothj@uni-muenster.de (J.R.)

**Keywords:** monocytes, macrophages, S100A8, S100A9, CD163, macrophage plasticity, inflammation, chronic inflammation, SIRS, COVID-19

## Abstract

Monocytes and macrophages are central players of the innate immune response and play a pivotal role in the regulation of inflammation. Thereby, they actively participate in all phases of the immune response, from initiating inflammation and triggering the adaptive immune response, through to the clearance of cell debris and resolution of inflammation. In this review, we described the mechanisms of monocyte and macrophage adaptation to rapidly changing microenvironmental conditions and discussed different forms of macrophage polarization depending on the environmental cues or pathophysiological condition. Therefore, special focus was placed on the tight regulation of the pro- and anti-inflammatory immune response, and the diverse functions of S100A8/S100A9 proteins and the scavenger receptor CD163 were highlighted, respectively. We paid special attention to the function of pro- and anti-inflammatory macrophages under pathological conditions.

## 1. Proinflammatory Immune Response

The human immune system is a complex interlinked system of cells, receptors and molecules that protect the human body from infections or tissue damage and orchestrate tissue healing and repair. The system has traditionally been divided into innate and adaptive. The adaptive immune system recognizes individual pathogens in a highly specific manner and for a long time it was assumed that it is solely responsible for the formation of immunological memory. It often offers the body lifetime immunity to re-infection with the same pathogen. The innate immune system on the other hand is the first line of defense against invading pathogens and relies on preserved receptors that detect the general characteristics of pathogens. These receptors are expressed on a variety of cells, facilitating rapid intervention in the event of an infection. It is believed that 99% of infections are responded to by the innate immune system [1]. The immune response can be divided into the following separate phases: homeostasis and surveillance; sensing and initiating the immune response (proinflammatory response); restoration of immune homeostasis and under certain physiological and pathological conditions, the generation of innate immune memory.

In addition to the tasks of immunological detection and the containment of infections, the innate immune system ensures the initiation of the adaptive immune response. The majority of cells of the innate immune system, mainly monocytes/macrophages, dendritic cells and granulocytes, derive from myeloid progenitor cells.

### 1.1. Inflammatory Monocytes and Macrophages

**Figure 1:**
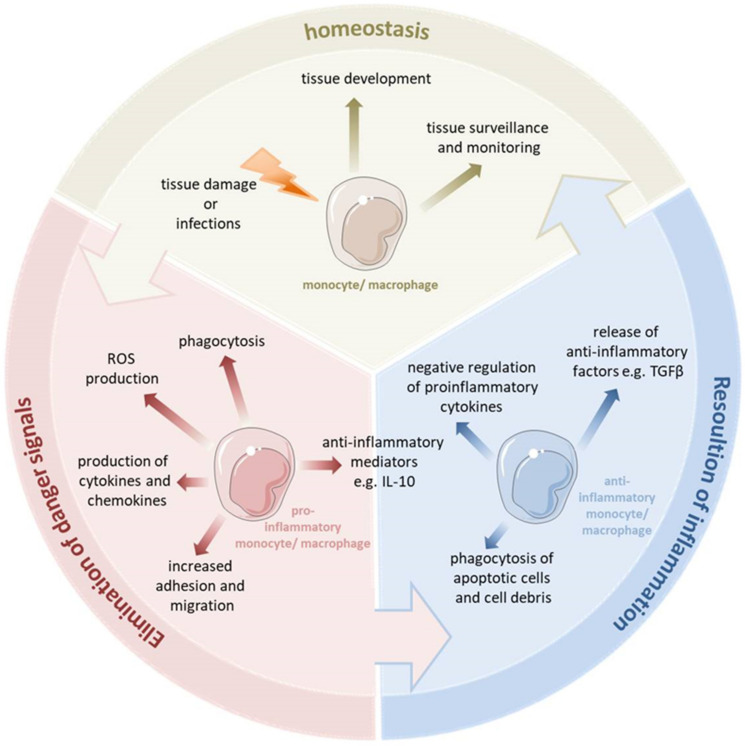
Monocytes and macrophages are a central component of the innate immune system. They are able to switch their phenotype from a homeostatic state to the proinflammatory state to eliminate pathogens and fight the inflammation. During uncomplicated inflammation, a switch from a proinflammatory to an anti-inflammatory phenotype occurs which enables the resolution of inflammation and the re-establishment of homeostasis. This enables the monocytes and macrophages to play diverse roles in the inflammatory response, both encouraging and discouraging this process. Depending on the kind of signal or pathophysiologic condition, monocytes and macrophages can undergo specific phenotypic polarization and thus acquire distinct functional phenotypes.

Monocytes and macrophages are a central component of the innate immune system and exert an important function in orchestrating inflammation [2]. They play a pivotal role not only in the generation of inflammatory mediators and in the regulation of the innate and adaptive immunity, but also contribute to the resolution of inflammation and the reestablishment of homeostasis (see Figure 1) [3,4]. Thus, the dysfunction of monocytes and macrophages is frequently involved in the pathophysiology of chronic infections and many severe sterile inflammatory and autoimmune diseases [5]. Monocytes account for 5–10% of all blood immune cells, and are bone-marrow-derived mononuclear cells with a life span of about 1–3 days [6,7]. In the steady state, they exert a homeostatic function and can differentiate into tissue macrophages. During an inflammatory process, monocytes are recruited to the site of inflammation and ultimately differentiate into inflammatory macrophages or dendritic cells [3]. 

Macrophages reside in every tissue of the body and exhibit a great functional diversity. They play a critical role in tissue development, the surveillance and monitoring of tissue changes, as well as in maintaining tissue homeostasis [8]. Many tissue macrophages are prenatally established during embryonic development from progenitors derived from the yolk sac or fetal liver and in the steady state they are maintained independently from the bone marrow-derived monocytes [9]. In contrast, dermal, heart and intestinal macrophages are first seeded by embryonic liver-derived progenitors but quickly after birth are replaced by monocytes derived from hematopoietic stem cells [10]. Tissue resident macrophages show a proliferative potential and a self-renewal capacity. The bone marrow-derived cells can contribute to the macrophage pool in inflammatory infiltrates and can replace tissue resident macrophages of an embryonic origin, e.g., after severe inflammation [11]. Tissue macrophages can be affected by a variety of factors to change their phenotype and thus their function. Monocytes and macrophages are considered particularly plastic. They have several different phenotypic states and, depending on the tissue type and environmental cues, they can acquire distinct functional phenotypes. They are able to switch their phenotype from a basal state (homeostatic functions) to the proinflammatory state (eliminating pathogen and battling the inflammation). During uncomplicated inflammation, a kind of switch from a proinflammatory to an anti-inflammatory/pro-resolving phenotype occurs, thereby promoting the resolution of inflammation and the re-establishment of homeostasis. This enables the monocytes and macrophages to play diverse roles in the inflammatory response, both encouraging and discouraging this process. Depending on the different environmental signals or various pathophysiologic conditions, monocytes and macrophages can undergo different forms of phenotypic polarization and thus acquire distinct functional phenotypes. 

The inflammatory response triggered by an infection or tissue damage involves the coordination of a multitude of cellular and molecular events. Pathogen recognition is considered as the most critical step for eliciting the adequate immune response during infection. Monocyte and macrophage activation and polarization are initiated through the recognition of pathogen- and tissue damage-associated conserved molecular motifs through pattern recognition receptors (PRRs) [12].

PRRs are germline-encoded host sensors that recognize the following two classes of molecules: pathogen-associated molecular patterns (PAMPs) which are evolutionarily conserved structures associated with pathogens such as viruses, bacteria, fungi and parasites and damage-associated molecular patterns (DAMPs), which are exposed in damaged host tissues. Currently, four different families of PRRs have been identified. These families include transmembrane receptors such as the Toll-like receptors (TLRs) and C-type lectin receptors (CLRs), as well as cytoplasmic proteins such as the Retinoic acid-inducible gene (RIG)-I-like receptors (RLRs) and NOD-like receptors (NLRs). With the exception of some NLRs, the sensing of PAMPs or DAMPs by PRRs upregulates the transcription of genes involved in inflammatory responses in monocytes and macrophages. The processes activated following the engagement of PRRs are rapid, induce conserved inflammatory patterns and include responses such as phagocytosis, cell locomotion, killing of pathogens or cells, and cytokine production. These innate immune mechanisms of monocytes and macrophages make them very effective in eliminating invading pathogens [13].

Among the PRRs, the TLR family has a unique capacity to sense the initial infection and induce an adequate inflammatory response [14]. TLRs are characterized by N-terminal leucine-rich repeats (LRRs) and a transmembrane region followed by a cytoplasmic Toll/IL-1R homology (TIR) domain. In humans, ten different TLRs have been identified. One of the most investigated TLRs present on the surface of monocytes and macrophages is TLR4, which is involved in the detection of Gram-negative bacteria and their associated endotoxins (e.g., Lipopolysaccharide, LPS), whereas lipoteichoic acid (LTA), peptidoglycan (PGN) and Pam3Cys of Gram-positive bacteria are recognized by TLR2 [15,16]. TLR signaling pathways consist, of at least a MyD88 (Myeloid differentiation primary response gene (88))-dependent pathway that is common to all TLRs and a MyD88-independent pathway that is peculiar to the TLR3 and TLR4 signaling pathways [17]. Endotoxin, or lipopolysaccharide (LPS), is an effective trigger of the inflammatory response during infection with Gram-negative bacteria and a very potent inducer of proinflammatory responses, especially in phagocytes. The activation of cells by LPS is initiated by an interaction with TLR4 and the formation of an LPS-binding complex with CD14 and MD-2 on the cell surface. Subsequently, the adaptor molecules MyD88 and TRIF (TIR-domain-containing adapter-inducing interferon-β) are recruited to the intracellular TLR4 domain resulting in the activation of either the “MyD88-dependent” or the “MyD88-independent” pathway in monocytes and macrophages.

In monocytes and macrophages, the “MyD88-dependent” pathway triggers the activation of downstream kinases, such as IL-1R-associated kinase-1 (IRAK-1), IRAK-4, phosphoinositide 3-kinase (PI3K), and mitogen-activated protein kinases (MAPK), and ultimately results in the liberation of the cytoplasmic nuclear factor ‘kappa-light-chain-enhancer’ of activated B-cells (NF-κB), its translocation to the nucleus and the subsequent transcription of inflammatory cytokines such as TNF-α, IL-6 and IL-1β. These cytokines promote the resulting immune response and induce the prolonged survival of monocytes [18]. The switching on of the “MyD88-independent” pathway in monocytes and macrophages leads to an activation of the transcription factor interferon regulatory factor 3 (IRF-3), and thereby induces interferon beta (IFN-β), finally resulting in the activation of several IFN-inducible genes [19,20,21]. Thus, macrophage activation leads to the induction of several potent mechanisms such as the production of ROS (reactive oxygen species), NO (nitrogen oxide) and the release of several cytokines to kill pathogens and combat the infection efficiently.

Over the last years, there has been growing evidence that TLRs play an important role not only in the recognition of pathogens by monocytes and macrophages but also in the sensing of DAMPs such as S100A8/S100A9, HMGB1 (high mobility group box protein-1), heat-shock proteins, uric acid and DNA. It has also been frequently reported that inflammatory processes induced by the activation of the TLR system via microbial products can be additionally modulated by endogenous ligands of TLR4. 

In this review, we described the function of monocytes and macrophages in the pro- and anti-inflammatory immune responses with special attention paid to the interplay between these two competing events and particular focus on S100 proteins and scavenger receptor CD163 as markers characteristic for the pro- and anti-inflammatory phenotype of monocytes and macrophages, respectively.

### 1.2. S100-Alarmins: Potent Effector Proteins of Inflammatory Macrophages

The most abundantly expressed DAMPs by monocytes and neutrophils are S100A8 and S100A9, which are proteins that belong to the group of alarmins and form about 5% of the soluble cytosol content in monocytes [22].

S100A8 and S100A9 are known to form non-covalently associated heterodimeric complexes (also called calprotectin) which represent the physiologically relevant forms of these proteins. S100A8, S100A9 and the S100A8-S100A9 complex are highly released in various inflammatory diseases locally and systemically and extracellular S100A8 and S100A9 show proinflammatory activities on many cell types, e.g., endothelial cells, phagocytes, lymphocytes or osteoclasts [23,24,25,26]. Interestingly, S100A8/S100A9 have been described to specifically interact with the TLR4-CD14-MD2 complex, thus representing inflammatory components that amplify phagocyte activation during the sepsis upstream of TNF-α-dependent effects [27]. They are released during many inflammatory processes in humans including sepsis and endotoxemia and they have been demonstrated to promote sterile inflammation in the absence of any microbial trigger as well [24,28,29,30]. Thus, on the one hand, these alarmins play a key role in the initial host defence against many infections and represent an important factor in orchestrating coordinated immune reactions. On the other hand, an uncontrolled and excessive release of these alarmins leads to an overwhelming inflammatory response and contributes to the dysregulated processes seen in many inflammatory, allergic and autoimmune conditions [25,30]. In addition to these proinflammatory functions, the prolonged stimulation of cells with S100A8 and S100A9 can induce hypo-responsiveness in monocytes and macrophages similar to the well-known endotoxin tolerance and thereby subsequently trigger immune paralysis, which is the major risk factor for enhanced morbidity and mortality during sepsis and SIRS (systemic inflammatory response syndrome) [29].

## 2. Anti-Inflammatory Immune Response

More than 30 years ago, it was shown that inflammatory processes show a sequence of infiltration of different macrophage populations—an activated subtype in the early phase characterized by the expression of S100A8 and S100A9, followed by an alternatively activated or anti-inflammatory macrophage population expressing scavenger receptor CD163 [31,32]. Macrophages can thus exhibit pro- and anti-inflammatory properties depending on the stage of the disease and signals they receive from the microenvironment. This ability of macrophages to rapidly adjust to changing microenvironmental conditions and to change their phenotype and function, a phenomenon known as macrophage plasticity, is now widely accepted and underlies macrophage polarization [33]. Interestingly, it was already known at that time that the plasticity of macrophages and the spectrum of macrophage activation are much greater than a simple separation into pro- and anti-inflammatory populations and rather a broad and complex spectrum of activated and anti-inflammatory phenotypes exists. 

Consequently, the still broadly accepted M1 and M2 paradigm of macrophage activation cannot fully explain the diversity of macrophage plasticity and a clear M1/M2 distinction is a gross oversimplification. This simple dichotomy has led to the establishment of markers belonging to M1 or M2 macrophages and the presence of markers associated with inflammation automatically leads to the grouping of the macrophage as M1. Similarly, if the marker is associated with the suppression of the immune response in this classification system, the macrophage is classified as M2. Nowadays, we know that macrophages activated in response to infection exhibit a proinflammatory phenotype. However, there is not one common phenotype for all inflammatory activated macrophages. It depends more on the macrophage origin, the tissue location and especially on the pathogen inducing macrophage activation and thus can lead to various activated phenotypes. The same is true for M2 macrophages. Despite the categorization of M2 macrophages into different subclasses (e.g., M2a, M2b, M2c and M2d) based on the nature of the inducing agent and expressed markers, M2 classification does not reflect the entire spectrum of anti-inflammatory macrophage phenotypes either.

Both activated and anti-inflammatory macrophages are closely related to inflammatory responses, among which the first are mainly involved in proinflammatory responses and the latter are mainly involved in anti-inflammatory and pro-resolving responses [33]. The classical activation of monocytes and macrophages takes place in response to very strong signals and is usually relatively short-term. Microbial products or proinflammatory cytokines (IFNγ or TNFα) induce the “proinflammatory” phenotype. These proinflammatory activated monocytes and macrophages constitute the first line of defense against invading pathogens, produce mainly IL-1β, IL-6, IL-12, IL-23, TNFα, NO and ROS, facilitate complement-mediated phagocytosis and promote the Th1 response. However, since several pathogens developed mechanisms to interfere with macrophage activation and blunt the proinflammatory activation, the proinflammatory phenotype of activated macrophages may significantly differ depending on the sort of invading pathogens and infection [34]. The duration of the activation phase depends on the balance between the capacity of the pathogens to survive, and the efficiency of the monocytes and macrophages to eliminate them, and ends with the cessation of stimuli. When macrophages can control the infection and remove the pathogens, the second phase of inflammation can be initiated to resolve the inflammation and to return the tissue to a homeostatic status. The effective termination of infection and inflammation requires the switch of the effector functions of macrophages from proinflammatory to anti-inflammatory, and the macrophage polarization dynamics are critical for terminating the proinflammatory response. This kind of switch between states of macrophage polarization is necessary to terminate the inflammatory response and reestablish homeostasis, and is observed in the course of many inflammatory diseases [35,36]. The release of anti-inflammatory and reparative mediators such as IL-4, IL-13, glucocorticoids, IL-10 or TGFβ, lipid mediators such as lipoxins, resolvins or protectins predominates in this phase and promotes the skewing of the macrophage phenotype towards anti-inflammatory and pro-resolution properties [37,38,39]. Anti-inflammatorily activated monocytes are most often triggered via persistent stimulation. Anti-inflammatory or pro-resolution monocytes and macrophages are characterized by the expression of CD204 (macrophages scavenger receptor type 1, MSR1), CD206 (mannose receptor) and arginase 1 (ARG1). These molecules not only determine the phenotype of anti-inflammatory macrophages but also contribute to the functional status of these cells by participating in the pattern recognition of pathogen infection, scavenging of unwanted mannoglycoproteins or the elimination of nitrogen by catalyzing arginine hydrolysis to urea and ornithine, respectively [40,41,42]. Anti-inflammatory monocytes and macrophages exhibit a broad spectrum of biological responses, e.g., they negatively regulate proinflammatory cytokines and release the anti-inflammatory factors such as IL-10 or TGFβ, orchestrate the resolution of inflammation, phagocytize apoptotic cells, coordinate tissue integrity but also promote tumor growth [43,44]. Anti-inflammatory macrophages promote wound healing, tissue repair and resolve inflammation [45]. 

The inflammatory process induces oxidative stress which is characterized by high levels of ROS overwhelming the buffering capacity of cellular antioxidants. Thus, inflammation and oxidative stress are closely intertwined. Inflammatory-activated macrophages are very efficient at recognizing, phagocytosing and killing invading pathogens in phagolysosomes. The bactericidal action of inflammatory polarized macrophages relies mostly on reactive nitrogen species (RNS) and ROS production, which in macrophages is mainly mediated by the Nox2 gene. Oxidative burst in inflammatory activated macrophages is necessary for the efficient killing of the invading pathogen and a positive loop exists between phagocytosis and ROS production—Nox2 dependent production of ROS is required for phagocytosis and has also been reported to increase the phagocytic activity of macrophages [46]. Thus, ROS production is usually associated with the activation and function of inflammatory activated rather than anti-inflammatory macrophages. In line with this, depending on the intracellular content of glutathione, which is a major intracellular antioxidant, the inflammatory and anti-inflammatory macrophages are characterized as oxidative and reductive, respectively [47]. However, despite having a lower ROS level, anti-inflammatory macrophages require ROS for their proper polarization. The inhibition of ROS generation, especially O^2−^, affects the polarization of anti-inflammatory, but not inflammatory-activated macrophages [48,49]. ROS is pivotal not only for the polarization of anti-inflammatory macrophages but serves also as an important factor in maintaining the undifferentiated state of myeloid-derived suppressor cells (MDSCs). It was previously reported that the scavenging of ROS, especially H_2_O_2_ with catalase, induced differentiation of MDSCs into macrophages and dendritic cells [49,50]. Additionally, ROS play a critical role in the clearance of apoptotic cells by anti-inflammatory and resolution macrophages. The early stage of apoptotic cell clearance is associated with an increase in ROS, which is then followed by the attenuation of oxidative burst. Similarly, pro-resolving lipid mediators produced at later stages of inflammation have been shown to inactivate Nox2 and inhibit ROS production after the engulfment of apoptotic cells [50,51]. Therefore, ROS and oxidative stress are involved in the regulation of macrophage polarization and function and may play a critical role in the progression of many diseases. Thus, macrophages are very important players regulating all phases of the immune response. From initiating inflammation and triggering the adaptive immune response through to the clearing of cell debris and resolving inflammation, they actively participate in all phases of immune response and constitute a bridge between all these processes. Under normal circumstances, the inflammatory response is temporal and resolves once the threat has passed. However, in some cases, acute inflammation does not end with a full resolution and instead leads to a state of low-grade systemic chronic inflammation. 

**Figure 2:**
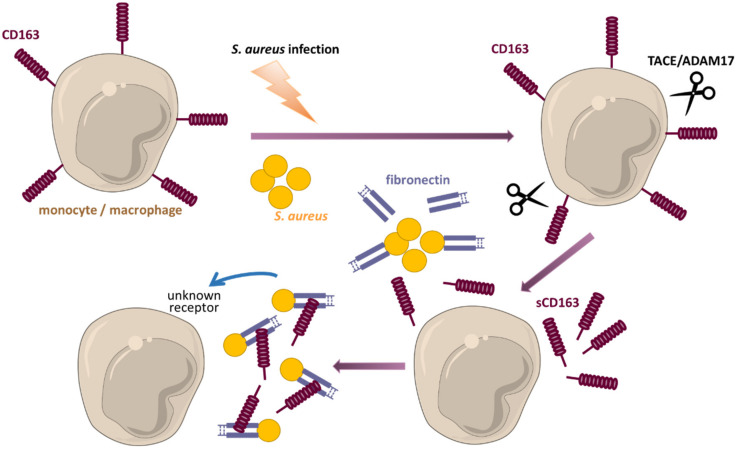
sCD163 modulates the host defense against staphylococcal infections. During sepsis membrane bound CD163 undergoes ectodomain shedding that leads to the generation of the soluble form of CD163 (sCD163). This process is mediated via cleavage by metalloproteinase TACE/ADAM17. *S. aureus* adheres to host tissue by binding of fibronectin. Once released, sCD163 also recognizes and binds to fibronectin, which then acts as a bridge between sCD163 and the staphylococci. This binding promotes the effective clearance (enhanced phagocytosis and killing) of *S. aureus* by professional phagocytes, while dampening the induction of proinflammatory cytokines and subsequently protecting the host from overwhelming inflammation.

One of the strongest endogenous regulators of inflammation are glucocorticoids (GCs). GCs represent also the most frequently employed drugs for short-term suppression of acute and chronic inflammatory diseases. Under physiological conditions, these hormones are released according to a circadian rhythm and regulate many physiological processes such as systemic fuel metabolism, water and electrolyte balance, the immune system and stress response [52]. GCs are also synthesized and secreted under stressful conditions, including infections and inflammation, and have an important role in regulating inflammatory processes as well as contribute to restoring of homeostasis. They act on all cells of the immune system including monocytes and macrophages. Classically activated macrophages are known to have an elevated expression of 11β-hydroxysteroid dehydrogenase (11β-HSD1), an enzyme favouring the activation of cortisol from cortisone and thereby increasing locally the effects of endogenous GCs [53]. GCs have been supposed to simply inhibit proinflammatory activation of macrophages and promote the differentiation of monocytes and macrophages towards an anti-inflammatory phenotype [54]. Endogenously derived GCs boost the capacity of anti-inflammatory activated (anti-inflammatory, wound healing, repair) macrophages to clear apoptotic cells and cells debris. Prolonged GCs treatment results in the differentiation of monocytes and macrophages with anti-inflammatory and pro-resolution properties that are characterized by the strong expression of the scavenger receptor CD163 [37,55]. Interestingly, this phenotype exhibits increased rather than decreased capacities to fight bacterial infections together with strong immunomodulatory properties protecting from overwhelming inflammation (see below) [56,57]. Thus, CD163 expression is a characteristic feature of macrophages that differentiate to an anti-inflammatory phenotype as well as of macrophages exerting regulatory properties. Accordingly, CD163^+^ macrophages are found in the resolution phase of inflammatory lesions and in wound healing tissues that again implicates an involvement of this molecule and CD163^+^ macrophages in the downregulation of the inflammatory response [32,58]. In steady state, the function of CD163^+^ macrophages is essentially a homeostatic one and strictly related to the binding and scavenging of toxic hemoglobin-haptoglobin complexes [59]. The interaction of hemoglobin-haptoglobin, with CD163 leads to the release of IL-10 and carbon monoxide (CO), which exert very strong anti-inflammatory effects. Significantly higher proportions of CD163^+^ circulating monocytes has been reported in patients undergoing elective coronary artery bypass graft surgery 24 h after the onset of bypass in parallel to production of higher amounts of IL-10 and heme oxygenase-1 (HO-1) indicating start of resolution of inflammation in response to bypass surgery [60]. CD163 deficient mice have shown a hindered anti-inflammatory response during the course of collagen-induced arthritis, as compared to wild type mice, indicating the pivotal role of CD163 and CD163^+^ macrophages in limiting arthritis progression [61]. Further, an increase in anti-inflammatory CD163^+^ macrophages has been also observed in the reparative and healing phase of cutaneous arteritis [62]. CD163^+^ anti-inflammatory macrophages have been reported to display an increased uptake of *S. aureus* and *E. coli* as well as latex beads [63,64]. Moreover, soluble CD163, which is shed from the surface of monocytes and macrophages upon activation, has been demonstrated to promote effective clearance of *S. aureus*, while dampening the induction of proinflammatory cytokines and subsequently protecting the host from overwhelming inflammation (see Figure 2) [56,57]. Furthermore, a recently described population of resident macrophages in the bone marrow and the spleen of wild type mice, which is characterized by a high CD163 expression and the origin of which depends on the transcription factor IRF8, exerts the gene expression pattern that partially overlaps to that of alternatively activated macrophages. This population has been shown to prevent overwhelming inflammation on the one hand but to effectively control *S. aureus* infections on the other hand [55]. Thus, targeting of macrophage polarization and modulation of macrophage polarity towards anti-inflammatory and pro-resolving phenotypes may be a promising approach for the treatment of many chronic inflammatory and infectious diseases.

## 3. The Yin and Yang of Pro- and Anti-Inflammatory Macrophages under Pathological Conditions

Overwhelming and chronic inflammation continue to be one of the biggest health problems world-wide. Thereby the patient’s recovery and outcome are highly determined by the restoration of a homeostatic balance between pro- and anti-inflammatory responses. Current therapies used for the treatment of most chronic inflammatory diseases are generally based on the concept that chronic inflammation is caused by an overwhelming inflammatory process but not due to the inadequate or insufficient regulatory and anti-inflammatory response or even an excessive immune suppression. Thereby common therapies for chronic inflammatory diseases rely on anti-inflammatory agents (e.g., methotrexate, glucocorticoids and anti-cytokine therapies) and thus dampen the unwanted inflammation instead to target regulatory mechanisms and rebalance the immune response. Since most inflammatory mechanisms are still not well understood, even modern therapies can rather treat the symptoms but not the causes and rarely induce long-lasting remission in chronic inflammatory diseases.

### 3.1. Macrophages in SIRS: A Prototype of Overwhelming Inflammation

The recognition of PAMPs or DAMPs is a potent trigger of macrophage-mediated inflammation. However, under distinct pathological conditions a prolonged exposure to these signals induces a state of tolerance that reprograms the inflammatory response of macrophages and results in an altered inflammatory cytokine production.

Sepsis is one of the leading causes of deaths world-wide and is still associated with a mortality rate of 25–30%. Currently sepsis is defined as a life-threatening organ dysfunction that is caused by a dysregulated host immune response to infection [65]. The innate immunity plays a direct role in the development of sepsis and is crucial for the activation and modulation of later antigen-specific adaptive immune responses. The sepsis syndrome often shows a biphasic course. In the first phase, the body’s own regulatory capacity fails and cells of the innate immune system (neutrophils and monocytes) show an uncontrolled release of proinflammatory cytokines such as TNFα, IL-6 and IL-1β. This may result in a “cytokine storm” that produces overwhelming inflammation, which can lead to blood pressure collapse, coagulation abnormalities and ultimately organ failure and death [66,67,68,69]. However, this process causes an excessive anti-inflammatory backlash that leads to immune paralysis. Therefore, patients who survive the cytokine storm may die from sepsis-related immunosuppression and an associated insufficient defense against secondary infections [69]. Most deaths occur nowadays during the stage of immune paralysis in the later phase of sepsis. The mechanism leading to the emergence of this immunosuppressive phase is called endotoxin tolerance. Endotoxin tolerance describes the reduced release of proinflammatory cytokines as a result of previous exposure to an immunostimulatory factor [70]. Immune cells that were previously in contact with low endotoxin doses and were subsequently stimulated with endotoxin revealed a significantly reduced inflammatory response compared to naive cells. This is characterized by a decreased expression and secretion of proinflammatory cytokines such as IL-6, IL-1β and TNFα [20,71]. Genes, such as the primary anti-inflammatory cytokine IL-10, TGFβ or the negative regulator IRAK-M are not generally suppressed, so no general down-regulation of LPS-induced genes can be assumed in endotoxin-tolerant cells [72,73]. Endotoxin tolerance can instead be seen as a form of genetic reprogramming resulting in an immunological memory of the innate immune response, a complex mechanism resulting in an altered immune response to a repeated inflammatory stimulus [20,74].

An immunosuppressive phase was also described for neutrophils and dendritic cells, but monocytes and macrophages are mostly responsible for the development of endotoxin tolerance in vivo [75]. Tolerant monocytes exhibit increased phagocytic activity and an increased expression of scavenger and lectin receptors, but show a significantly reduced ability for antigen presentation [71,75,76]. 

The exact mechanism of endotoxin tolerance is still largely unknown but appears to be caused by changes in the cellular response arising from alterations in signal transduction pathways [20,77]. LPS is known to initiate multiple signal transduction cascades downstream of TLR4 and endotoxin tolerance-associated defects in TLR4 signaling have been observed at the level of the receptor, adaptors, signaling molecules, and transcription factors. Thereby, the described defects map to both the MyD88-dependent and the MyD88-independent signaling cascade. A decreased TLR4–MyD88 complex formation, an impairment of IRAK-1 activity, and defects in the activation of mitogen-activated protein kinases (MAPKs) and NF-κB have been described [20]. In contrast, the MyD88-independent pathway has been demonstrated to remain functional in endotoxin tolerance and an overexpression of TRIF-induced genes such as IFNB has been described [78,79]. This type I interferon was shown to be an important regulator of endotoxin tolerance, by inducing the upregulation of IL-10 [80]. Furthermore, an up regulation of so-called negative regulators of the TLR-signaling pathway, for example A20, IRAK-M, SOCS1/SOCS3 and BCL3, has been reported [75]. Monocytes from sepsis patients were described to over-express transcriptional inactive NF-κB dimers [81]. Transcriptome studies in monocytes/macrophages revealed that these cells undergo major gene reprogramming during endotoxin tolerance. An important mechanism in this context is the TLR-dependent induction of epigenetic chromatin and DNA modifications. In particular, histone modifications play fundamental roles in regulating gene expression and a huge catalogue of histone modifications has been described ranging from lysine/arginine methylation, lysine acetylation and serine/threonine/tyrosine phosphorylation. Several studies described that the induction of inflammatory genes is marked by the acetylation of histone H4 (H4K8), methylation at H3K4 and acetylation at H3K9 and H3K14 [82,83,84], whereas the silencing of subsets of these genes is associated with a methylation of H3K27 and H3K9 [84,85]. LPS was also shown to promote gene silencing by inducing the histone methylation of gene promoters [76]. However, a complete functional understanding of histone modifications in endotoxin tolerance is still lacking. In addition, the microRNA-mediated regulation of the endotoxin response emerged as an important post-transcriptional regulatory mechanism for gene expression. These non-coding RNAs, consisting of 17–27 nucleotides, can attach to complementary mRNA sequences and thereby induce post-transcriptional mRNA degradation or inhibit translation [86]. Proinflammatory stimuli such as LPS, TNFα, and IL-1β induce the expression of specific microRNAs that affect the TLR4 and IL-1 receptor signaling pathways in monocytes and macrophages. Two of the prominent microRNAs involved in inducing endotoxin tolerance are miRNA146 and miRNA155, both of which have been shown to post transcriptionally regulate the expression level of signaling molecules of the TLR4 or NF-κB signaling pathway (e.g., IRAK-1, TRAF6 and IKKe) [87,88,89,90]. 

The induction of endotoxin tolerance also alters the metabolism of monocytes and macrophages. The stimulation of macrophages with either LPS or cytokines (e.g., TNFα, IL-6, IFNγ), as well as the induction of hypoxia, induces their classical activation, which is associated with a metabolic shift towards aerobic glycolysis (called Warburg effect), the production of ROS, the disruption of the tricarboxylic acid (TCA) cycle and the inhibition of oxidative phosphorylation [91,92,93]. LPS stimulation induces glutamine metabolism and the production of α-ketoglutarate and succinate in macrophages. Therefore, succinate augments the expression of IL-1β by stabilizing HIF1α, whereas α-ketoglutarate induces the opposite effect. However, an alternative activation of monocytes by IL-4, IL-13 or IL-10 stimulation leads to an inhibition of both proinflammatory cytokine production and glycolysis, but increases oxidative phosphorylation, the uptake of fatty acids and the expression of fatty acid transporters such as scavenger receptors and CD36 [92]. All in all, there exists a complex interplay between metabolic function, cytokine production, and tolerization, although it remains unclear whether tolerance regulates metabolism or vice versa. 

However, it is not only pathogens that cause the secondary stage of immune paralysis. The characteristic biphasic immune response can also be seen in patients with non-infectious systemic inflammatory response syndrome (SIRS). SIRS is a clinical picture that is similar to sepsis but lacks the proof of an infection with pathogens [94]. SIRS affects most patients with a severe burn or trauma, with or without the presence of an infection. Interestingly, hypo-responsiveness can also be triggered under sterile conditions by endogenous TLR4 ligands, such as DAMPs. DAMPs are known to induce both an excessive immune response and immune paralysis. In trauma patients, in patients with severe burns or after surgical interventions, a massive release of DAMPs was previously demonstrated [95,96,97,98]. Two prominent DAMPs that play a crucial role in the pathogenesis of SIRS are the S100 proteins S100A8 and S100A9. Polytrauma and burn trauma patients initially present with high serum concentrations of these S100 proteins. In burn trauma patients, the measured S100A8/S100A9 concentrations significantly correlate with the percentage of at least second-degree burned total body surface area (TBSA) and survivors show significantly lower S100A8/S100A9 concentrations than non-survivors [29]. Increased S100A8/S100A9 concentrations can also be found in patients after cardiopulmonary bypass surgery (CBS). CBS induces major sterile stress during surgery and extracorporeal circulation, and the inflammatory response induced during CBS is implicated in many of the postoperative clinical problems faced by these patients [99]. CBS patients show already significantly increased serum levels of S100A8/S100A9 at the time of cardiopulmonary bypass removal, which then remain elevated for 6 days [30]. 

**Figure 3:**
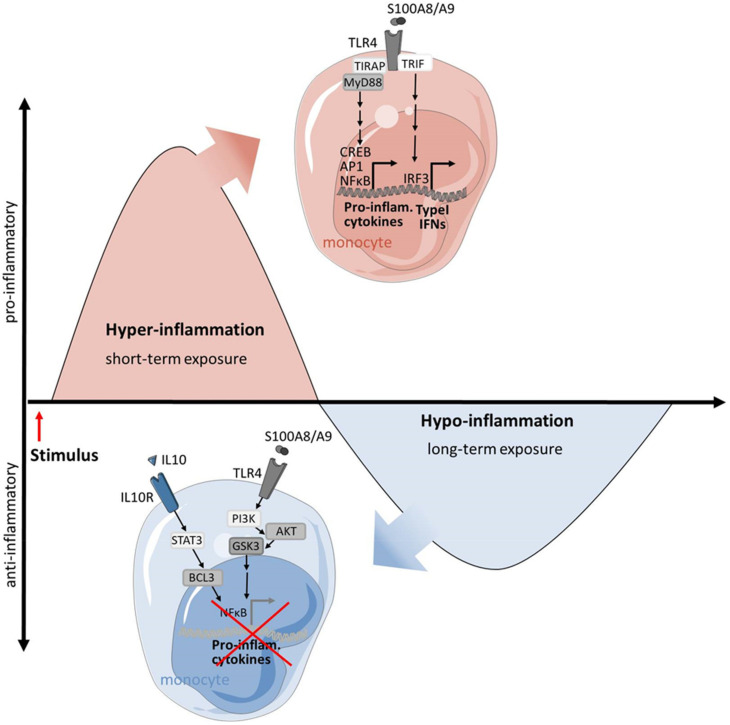
S100A8 and S100A9 are well known ligands of TLR4 and induce the expression of proinflammatory cytokines and type I interferons in monocytes and macrophages. Therefore, they function as amplifiers of phagocyte activation during the early hyper-inflammatory state of SIRS. However, long-term stimulation with S100A8/S100A9 has a regulatory role in promoting phagocyte hypo-responsiveness to subsequent inflammatory stimulation. It was shown that prolonged stimulation with low doses of S100A8 and S100A9 leads to an activation of the phosphatidylinositol 3-kinase/AKT/GSK-3β pathway which interferes with the NF-κB–driven expression of proinflammatory cytokines. This inhibition of cytokine production is further enhanced by secondary IL-10 triggered activation of STAT3 and BCL-3.

Interestingly, the TLR4 ligands S100A8/S100A9 do not only function as amplifiers of phagocyte activation during SIRS, they also play a regulatory role in promoting phagocyte hypo-responsiveness to subsequent inflammatory stimulation (see Figure 3) [27,29,100]. Therefore, the S100A8/S100A9-induced hypo-inflammation resembles the classical LPS-induced tolerance. Bioinformatics analyses of genome-wide transcriptomes of S100A8/S100A9- and LPS- pre-stimulated human monocytes revealed that S100A8/S100A9 pre-stimulation induces an almost identical response pattern in human monocytes as LPS [29]. In vitro pre-stimulations of monocytes with low doses of S100A8/S100A9 result in a significantly decreased cytokine release in response to subsequent TLR4 stimulation. Furthermore, ex vivo stimulations of monocytes from CBS patients demonstrated that these monocytes exhibit a state of hypo-responsiveness that is characterized by a remarkably reduced TNFα expression and secretion [30]. Accordingly, pre-treatment with S100A8/S100A9 protected mice in an experimental endotoxic shock model, similar to classically induced LPS tolerance [29,101]. All in all, these data demonstrate the high biological relevance of hypo-responsiveness that can be triggered under sterile conditions by endogenous TLR4 ligands, such as S100A8/S100A9.

However, signaling pathways that trigger the hypo-responsiveness of phagocytes in clinically relevant diseases are only barely understood. A recent study with CBS monocytes identified two main signaling cascades that are activated during the S100-induced tolerance of monocytes. On the one hand, prolonged stimulation with S100 proteins leads to an activation of the phosphatidylinositol 3-kinase/AKT/GSK-3β pathway which finally interferes with NF-κB-driven gene expression. On the other hand, interleukin-10 triggers the activation of transcription factors STAT3 and BCL-3. BCL-3 interferes with NF-kB activity and monocytes obtained from patients with dominant-negative STAT3 mutations are prevented from possessing S100-induced tolerance. In summary, these results underline the importance of sterile triggers such as S100 proteins for both phases of the immune response in SIRS, the induction of the primary, proinflammatory phase and the suppression of the subsequent secondary hypo-inflammatory phase [30].

Very little is known regarding the role of CD163^+^ monocytes and macrophages in sepsis. In response to activation, CD163 undergoes shedding from the surface of monocytes and macrophages by the metalloproteinase TACE/ADAM17, the enzyme also responsible for the cleavage of membrane-bound TNFα, which leads to the release of sCD163 plasma protein [102]. The serum levels of sCD163 positively correlate with the severity of many infectious and inflammatory diseases. The rapidly increased serum level of sCD163 mediated by ADAM17 was observed in experimental endotoxemia induced in healthy volunteers by the injection of LPS [102,103]. Similarly, septic patients exhibited an increased serum level of sCD163 [104]. Thus, it is currently evident that sCD163 is a useful marker of monocyte and macrophage activation and a promising prognostic marker of comorbidity and mortality in sepsis [105,106]. However, whether these levels reflect the start of an anti-inflammatory regulatory phase is currently not clear.

### 3.2. Pro- and Anti-Inflammatory Macrophages in SARS-CoV-2 Infection

The highly contagious Coronavirus disease 2019 (COVID-19) is caused by the SARS-CoV-2 (severe acute respiratory syndrome coronavirus 2) virus. Signs and symptoms of COVID-19 include respiratory symptoms such as cough and shortness of breath and fever. In more severe cases, COVID-19 can cause pneumonia, severe respiratory distress and in some cases multi-organ failure and death. The activation of innate immune cells is a major factor in severe courses [107]. Clinical studies revealed high S100A8 and S100A9 serum concentrations as the best predictive biomarkers for critical COVID-19 cases and mortality [108,109,110], indicating that these endogenous ligands of TLR4 amplify also the aberrant innate anti-viral response during SARS-CoV-2 infection [111]. However, the exact role of S100A8 and S100A9 during this infection is still under investigation and it has still to be clarified how both S100A8/S100A9 mechanistically contribute to the outcome of COVID-19.

In addition, elevated levels of sCD163 were detected in the serum of SARS-CoV-2 infected patients admitted to hospital. Peripheral blood monocytes isolated from patients affected by COVID-19 related pneumonia exhibited an increased expression of CD163 as well. All patients presented with an anti-inflammatory and immune-regulatory phenotype characterized by the reduced expression of CD80, CD86 but the enhanced expression of CD204, CD206 and PD-L1. Moreover, the expansion of these anti-inflammatory CD163^+^ monocytes was higher in the patients featuring a detectable SARS-CoV-2 plasma viral load and positively correlated with the levels of specific antibodies [112]. Similar observations were made when analyzing the lung tissues from patients infected with COVID-19. An investigation of the pulmonary immune responses and lung pathology in two cohorts of patients with COVID-19 acute respiratory distress syndrome (ARDS) revealed in the lung an accumulation of CD163^+^ monocyte-derived macrophages that acquired a pro-fibrotic transcriptional phenotype during COVID-19 ARDS and thus probably contributed to the exacerbated fibro-proliferative response in COVID-19-associated ARDS [113]. These results implicate that SARS-CoV-2 drives monocytes and macrophages that induce the anti-inflammatory phenotype of the host for the benefit of COVID-19 disease progression.

### 3.3. Macrophages in Chronic Inflammatory Conditions

Chronic inflammation is characterized by a continuous recruitment of monocytes and lymphocytes. In contrast to acute inflammatory responses, chronic inflammation may last weeks, months, or in some cases, even a lifetime. Chronic inflammation plays a key role in the development and progression of many clinically relevant diseases, e.g., in autoimmune diseases, allergies, chronic infections but also in cardiovascular diseases and cancer. 

Monocytes and macrophages are key cellular components involved in the development and regulation of numerous chronic inflammatory conditions such as cancer or autoimmune diseases such as rheumatoid arthritis, thus contributing to the pathogenesis of these disorders [114]. Proinflammatory-activated macrophages have been identified as important mediators in several chronic diseases including rheumatoid arthritis, multiple sclerosis or autoimmune hepatitis and the impaired phenotype transition of proinflammatory activated to anti-inflammatory monocytes and macrophages is a characteristic feature of chronic inflammatory disorders. Under normal circumstances the activity of macrophages switches in the course of inflammation from proinflammatory to anti-inflammatory to achieve homeostasis. However, persisting inflammation and incomplete resolution commonly result in chronic inflammation.

Up to now, more than 100 different autoimmune diseases have been described. These can be divided into organ-specific (e.g., type I diabetes) and systemic (e.g., rheumatoid arthritis (RA)) autoimmune diseases. RA is the most common inflammatory rheumatic disease and primarily affects the synovial tissue of joints, which over time results in cartilage and bone destruction. Synovitis in the RA joint is characterized by the persistent infiltration and accumulation of various immune cells, mainly monocytes and macrophages. Several soluble mediators released by these immune cells have been shown to correlate with RA progression and/or severity, including cytokines such as TNFα and IL-6, and alarmins such as S100A8 and S100A9 [25,26]. S100A8 and S100A9 were first described in the context of RA. Especially for synovial macrophages, increased S100A8/S100A9 expression levels as well as S100-induced activation were described [115,116,117]. Moreover, synovial macrophages isolated from inflamed RA joints were shown to secrete high levels of S100A8 and S100A9 which leads to the increased secretion of proinflammatory cytokines such as TNFα, IL-6 and IL-1ß [118]. However, S100A8 and S100A9 have also been shown to activate chondrocytes and osteoclasts and thereby promote cartilage and bone destruction [119,120]. All in all, S100A8 and S100A9 were considered as key effector molecules in the pathogenesis of RA. Moreover, several studies described S100A8 and S100A9 as valuable and predictive biomarkers for local disease activity due to their release at sites of inflammation. Accordingly, S100A8/S100A9 levels were significantly higher in the synovial fluid compared to the serum and correlate with local markers of joint inflammation but not with systemic parameters of disease activity [25]. Thus, the detection of the local S100 expression would even enhance their diagnostic potential. In recent years, an increased focus has been placed on experimental mouse models. Using Cy5.5-labelled S100A9 antibodies, disease activity and progression could be monitored in an experimental arthritis model [121]. In addition, a non-peptidic S100A9 tracer (CES271-Cy5.5) was developed and used for specific optical imaging in exemplary murine disease models [122,123]. Moreover, in a seronegative experimental arthritis model, local S100A8 was monitored in vivo with optical imaging using anti-S100A8-Cy7 tracers.

Lately, S100A8 and S100A9 have been increasingly used as in vivo imaging biomarkers in cancer [124]. Accordingly, the target-specific imaging of S100A8/A9 can be used to visualize the tumor-immune interaction. The optical in vivo imaging of S100A9 in tumor lesions helps in evaluating the tumor–host cell interaction and enables the estimation of the individual malignant potential [125,126].

All in all, these data identify S100A8 and S100A9 as local players of inflammation and potential targets for innovative treatment approaches. 

The soluble form of CD163 increases in the plasma of patients suffering from chronic inflammatory disorders [127]. In many chronic inflammatory diseases, CD163 is also upregulated at the sites of inflammation, thereby reflecting the accumulation of CD163^+^ macrophages as an important part of the chronic inflammatory response [128]. However, the role of CD163 in chronic inflammation has not been defined yet. The presence of CD163^+^ macrophages may either represent a special stage of inflammation or the CD163 expression on these macrophages is just a simple consequence of gene regulation that is insufficient to exert the full anti-inflammatory effects. Recently, it was shown that CD163^-/-^ mice revealed a predominant Th2 response, resulting in increased inflammation and joint destruction in a model of experimental collagen-induced arthritis. This confirmed the importance of CD163 as a regulator of chronic inflammation. However, the exact molecular mechanism still needs to be elucidated [61]. 

Chronic inflammation also modulates tumor growth and metastasis during cancer [129]. One of the hallmarks of cancer is the abnormal differentiation and function of myeloid cells including monocytes and macrophages. The accumulation of myeloid-derived suppressor cells (MDSCs) is commonly found in cancer. MDSCs represent a heterogenous population of relatively immature myeloid cells with a strong immunosuppressive activity. MDSCs with a monocytic origin are termed M-MDSCs and the major developmental factors for M-MDSCs are granulocyte–macrophage colony-stimulating factor (GM-CSF), macrophage colony-stimulating factor (M-CSF/CSF1), IL-6, IL-1β and adenosine signaling [130]. M-MDSCs are confirmed to be monocytes in morphology and phenotype but possess a distinct biochemical and metabolic profile. One of the important characteristics of M-MDSCs is their ability to suppress immune cells, and especially to inhibit T cells. A number of signaling pathways responsible for MDSC activity have been reported. However, the IL4Ra-STAT6 pathway seems to be particularly important for the suppressive functions of MDSCs. It mediates the activity of inducible arginase 1 (ARG1), TGF production and in cooperation with STAT1 and STAT3 it promotes the production of ROS and peroxynitrite [131]. Other molecules reported to be involved in the inhibitory activity of M-MDSCs are nitric oxide synthase (NOS2/iNOS) and IL-10 [132]. Thus, MDSCs are involved in the regulation of immune responses and their accumulation in cancer is associated with a poor clinical outcome. S100A8 and S100A9 proteins have also been reported to play an essential role in the generation, recruitment and accumulation of MDSCs and a lack of S100A8/S100A9 reduced the suppressive activity of MDSCs [133,134,135,136]. MDSCs have been frequently described in patients with various autoimmune disorders such as multiple sclerosis, RA and inflammatory bowel disease, in which elevated S100A8 and S100A9 have been reported to contribute to disease pathology [137]. In autoimmune diseases, M-MDSCs can exert a double-faced role with either beneficial or detrimental outcomes for the host. They can play protective and helpful roles in resolving inflammation and in the establishment of the immune homeostasis after infection or tissue injury by limiting excessive inflammatory processes due to restricting T cell function, inducing regulatory T cells and releasing immunosuppressive cytokines and other soluble mediators. However, the expansion of M-MDSCs can also negatively regulate immunity through, among other aspects, stimulating Th17 cell differentiation and facilitating the B cells’ response which promotes disease persistence [138,139,140]. Thus, the role of MDSCs in chronic and autoimmune diseases remains controversial. 

MDSCs present in tumor sites have been shown to differentiate into tumor-associated macrophages (TAMs), but are not the only source of TAMs [141,142]. It has also been shown that TAMs originate from bone marrow-derived CCR2^+^ monocyte precursors or spleen monocytes and that their differentiation depends on the Notch signaling pathway via RBP1 [143]. Phenotypically, TAMs can be distinguished from M-MDSCs by the increased expression of F4/80, low or intermediate expression of Ly6C, low or even undetectable expression of the S100A9 protein and increased IRF8 and M-CSF receptor (CD115) expression [144,145]. M-CSF can recruit monocytes to the tumor tissue where IL-4, IL-10, IL-13 and other factors present in the tumor microenvironment can induce the differentiation of monocytes into TAMs. TAMs represent a major compartment of the tumor microenvironment and play a pivotal role in tumor growth, metastatic dissemination and therapy failure. TAMs have been reported to promote angiogenesis, support tumor growth and especially inhibit the anti-tumor immune response [146]. Generally, TAMs are accepted to promote tumor progression using different mechanisms. They express a variety of factors stimulating tumor growth such as epithelial growth factor (EGF), hepatocyte growth factor (HGF) or epithelial growth ligands of the factor receptor (EGFR) family [147]. TAMs have also been reported to produce soluble factors such as matrix metalloproteinases (MMPs), serine proteases, cathepsins decomposing various components of extracellular matrix and thereby enabling the migration of tumor cells that promote metastasis [148,149]. TAMs promote angiogenesis by releasing proangiogenic factors such as BFGF, VEGF, IL-1, IL-8, TNFα, MMP-9, MMP-2, and NO [150]. TAMs can also negatively influence the immune response and downregulate the killing activity of NK and T cells against tumor, directly inhibit CD8^+^ T cells proliferation and also recruit Tregs in a CCL20-dependent manner, which further suppress the antitumor response of T cells [151]. TAMs generated from MDSCs have a higher expression of S100A8/S100A9 than TAMs generated from monocytes. A positive correlation between the immunogenicity of tumors and S100A8/S100A9 expression has also been reported and the overexpression of S100A9 resulted in a higher expression of markers characteristic of anti-inflammatory macrophages and a markedly stronger immune suppressive activity of TAMs. S100A8/S100A9 proteins released by phagocytes also contributed to the early recurrence of cancer and tumor invasion [152,153]. Moreover, the overexpression of S100A9 during macrophage differentiation promoted an anti-inflammatory (expression of ARG1 and CD206) phenotype in macrophages, even in the absence of tumors, and recapitulated the effect observed in TAMs [135]. One of the commonly used markers for the identification of TAMs in tumor tissues is CD68. CD68^+^ TAMs have also been reported to express CD163, CD204, CD206, ARG1 and IL-10, which are generally characteristic of anti-inflammatory macrophages. However, the phenotype of TAMs is not identical to that of anti-inflammatory monocytes, but there is rather an overlap between the molecular signature of TAMs and anti-inflammatory macrophages [154]. Moreover, TAMs are quite heterogeneous and TAMs found at different sites and histological types of cancer differ regarding the expression of many markers. In human ovarian cancer, the CD163^+^ CD204^+^ TAMs positively correlate with the histological grade of malignancy. In kidney cancer, malignant lymphoma or glioma, a higher expression of CD163 on TAMs strongly correlates with a poor clinical prognosis, but no correlation exists between the clinical prognosis and the number of CD204^+^ TAMs. In contrast, in esophageal cancer, CD204 is an indicator of poor outcome, whereas CD163 is not [155,156]. Thus, TAMs are an important component of the tumor microenvironment and a very attractive target for cancer immunotherapy.

## 4. Modulation of the Macrophage Response as Innovative Therapy

Current therapies used for the treatment of most chronic inflammatory diseases rely on anti-inflammatory agents and thus dampen the unwanted inflammation instead of targeting the regulatory mechanisms and rebalancing the immune response. In particular, the specific blockade of cytokines or of their downstream signaling pathways has experienced significant progress in the treatment of inflammatory diseases. However, these therapies show adverse side effects and long-lasting remission after stopping medication is generally not achieved. Thus, there is still an urgent need for innovative therapeutic approaches. A more promising and specific approach would therefore be to directly target monocyte/macrophage functions by inhibiting specific molecular targets, such as the S100 proteins S100A8/S100A9. As a result, S100 proteins themselves could be central in such treatments, or they could be used in a targeted manner to modulate the immune response. S100A8/S100A9 could be targeted by inhibiting their expression and secretion or by blocking their receptor interactions. Currently, there are only few data available demonstrating the effective blocking of S100-protein expression in vivo. However, the knock-out of S100A9 in mice points to its possible efficacy in the treatment of different inflammatory conditions and a peptide sequence of about 15 amino acids seems to be a molecular target for the inhibition of S100-TLR4 interactions [25,157]. In addition, the use of specific short hairpin RNAs was effective to hamper the expression of S100A8/S100A9 in human myeloid cells of myelodysplastic syndrome patients [158]. Interestingly a group of anti-inflammatory drugs, Q-compounds, have been demonstrated to block S100-mediated macrophage activation and to prevent the interaction of S100A8/S100A9 with TLR4 and RAGE [159,160]. The initial clinical data demonstrate that Q-compounds are effective in the treatment of multiple sclerosis and Crohn’s disease [161,162]. S100A8/S100A9 are specifically secreted via an alternative microtubule and active caspase-1 dependent secretion pathway [163]. Interestingly, in Familial Mediterranean Fever, an autoinflammatory disease characterized by extraordinarily high serum levels of S100A8/S100A9, the inhibition of microtubule polymerization by colchicine is used as first line medication [164]. Another therapeutic option in inflammatory diseases would be the targeting of PRRs such as TLR4 [165,166] and RAGE [167,168] by antibodies and inhibitors. However, blocking TLR4 presents the major risk of suppressing anti-microbial recognition.

Hypothetically, an interesting treatment option for inflammatory diseases would be to directly modulate patients’ immune responses by using S100 proteins. Prolonged exposure to S100-proteins shows immunomodulatory effects on phagocytes and induces immune tolerance [29]. Thus, blood-derived phagocytes that have specifically been modulated by S100A8/A9 treatment ex vivo could be re-transfused to reduce overwhelming immune responses in inflammatory diseases.

## 5. Conclusions

Monocytes and macrophages are a heterogeneous population of immune cells playing various functions in homeostasis and immune responses. The broad spectrum of macrophage activities and functions is strictly dependent on their plasticity that allows them to adapt their phenotypes to changing environmental signals and to modify their functional properties. A broad spectrum of macrophage phenotypes exists and it is very difficult to categorize them as strictly pro- and anti-inflammatory or regulatory. Depending on the source of macrophages (origin, tissue location) and the definition of activators (quantity and duration of exposure), different phenotypes emerge, which admittedly share many common characteristics but differ more or less in function. A better understanding of monocyte and macrophage behavior is thus crucial to deciphering the pathogenesis of systemic inflammation and chronic inflammatory diseases and to develop more effective therapies for their treatment.
